# Association between body fat percentage and H-type hypertension in postmenopausal women

**DOI:** 10.3389/fpubh.2022.950805

**Published:** 2022-07-22

**Authors:** Shihong Du, Xiuqin Hong, Yi Yang, Zihao Ding, Tong Yu

**Affiliations:** ^1^Clinical Epidemiology Research Office, The First Affiliated Hospital of Hunan Normal University, Changsha, China; ^2^Key Laboratory of Molecular Epidemiology, Hunan Normal University, Changsha, China; ^3^Department of Epidemiology and Statistics, College of Medicine, Hunan Normal University, Changsha, China; ^4^Research Department, The First Affiliated Hospital of Hunan Normal University, Changsha, China

**Keywords:** H-type hypertension, body fat percentage, obesity, postmenopausal women, cross-sectional study

## Abstract

**Background:**

Previous studies have explored the relationship between body fat percentage (BFP) and hypertension or homocysteine. However, evidence on the constancy of the association remains inconclusive in postmenopausal women. The aim of this study was to investigate the association between BFP and H-type hypertension in postmenopausal women.

**Methods:**

This cross-sectional study included 1,597 eligible female patients with hypertension. Homocysteine levels ≥10 mmol/L were defined as H-type hypertension. BFP was calculated by measuring patients' physical parameters. Subjects were divided into 4 groups according to quartiles of BFP (Q1: 33.4% or lower, Q2: 33.4–36.1%, Q3: 36.1–39.1%, Q4: >39.1%). We used restricted cubic spline regression models and logistic regression analysis to assess the relationship between BFP and H-type hypertension. Additional subgroup analysis was performed for this study.

**Results:**

Among 1,597 hypertensive patients, 955 (59.8%) participants had H-type hypertension. There were significant differences between the two groups in age, BMI, educational background, marital status, exercise status, drinking history, WC, TG, LDL, Scr, BUN, and eGFR (*P* < 0.05). The prevalence of H-type hypertension in the Q1 to Q4 groups was 24.9, 25.1, 24.9, and 25.1%, respectively. After adjusting for relevant factors, we found that the risk of H-type hypertension in the Q4 group had a significantly higher than the Q1 group (OR = 3.2, 95% CI: 1.3–7.5).

**Conclusion:**

BFP was positively associated with the risk of H-type hypertension in postmenopausal women. Postmenopausal women should control body fat to prevent hypertension.

## Introduction

H-type hypertension is essential hypertension associated with serum homocysteine (Hcy) levels >10 umol/L (1). H-type hypertension is found in 80.3% of hypertensive patients in China. Epidemiological studies have indicated that when hypertension and hyperhomocysteinemia (HHcy) coexist, the risk of cardiovascular and cerebrovascular illness increase (2). As a result, early intervention is critical in H-type hypertension patients.

In recent years, obesity has increasingly become one of the most prominent global health problems (3). A million-person epidemiological survey in China reported that more than 50% of the adult residents are overweight or obese (4). Recent evidence has examined the relationship between obesity and H-type hypertension, and being obese or overweight is associated with an increased risk of H-type hypertension (5). Body mass index (BMI) has been the most frequent indicator of obesity due to its simplicity of measurement and low cost, but whether BMI can explain obesity remains controversial (6). A possible explanation is that BMI only takes into account height and weight, not fat mass and lean body mass (7). Body fat percentage (BFP) is a more accurate indicator of body fat composition than BMI.BFP is defined as the ratio of body fat weight to total body weight and offers a more precise view of body fat composition (8). There is growing evidence that increased BFP is associated with an increased risk of developing hypertension, even in individuals with normal BMI (9). These findings suggest the possibility of BFP being a risk factor for hypertension.

Postmenopausal women are more exposed to cardiovascular diseases like hypertension and HHcy due to decreased estrogen (10). Previous studies suggested that postmenopausal women have a higher prevalence of H-type hypertension than both premenopausal women and men of the same age (11, 12). Furthermore, several studies have revealed that estrogen deficiency significantly increases BFP, resulting in an increased risk of hypertension in postmenopausal women (13). Although previous researchers have investigated the relationship between BFP and hypertension, this association remains unexplained in postmenopausal women (14). Therefore, this cross-sectional study aimed to investigate the relationship between BFP and the risk of H-type hypertension in postmenopausal as well as to provide the groundwork for protecting postmenopausal women from H-type hypertension.

## Materials and methods

### Material

#### Study population

This cross-sectional study was conducted at Hunan Provincial People's Hospital, China, from December 2018 to December 2020. Inclusion criteria:(1) postmenopausal women (menstruation stopped for more than 1 year); (2) subjects diagnosed with essential hypertension (5). Exclusion criteria: (1) subjects who had recently taken medications that may affect blood pressure and Hcy; (2) secondary hypertension caused by pheochromocytoma, sleep apnea syndrome, and so on; (3) subjects with language impairment, mental illness, etc. who could not reflect the situation or did not cooperate with the questionnaire.

The project plan got accredited by the medical ethics committee of Hunan Normal University (No. 034/2017). All participants signed informed consent before participating in this study.

#### Data collection and anthropometric

The demographic characteristics of each individual were collected by trained researchers through questionnaires. The questionnaires included age, education level, marital status, smoking history, alcohol consumption history, and exercise status.

Blood pressure was measured by professionally trained nurses. Patients were prohibited from strenuous exercise, smoking and coffee half an hour prior to blood pressure measurement, and 3 measurements were taken using an electronic sphygmomanometer with at least 5 min of rest each time. The average of the 3 values was calculated and documented. Five milliliter of fasting venous blood were collected from all study subjects using an anticoagulation tube. Triglycerides (TG), total cholesterol (TC), low-density lipoprotein cholesterol (LDL), high-density lipoprotein cholesterol (HDL), alanine aminotransferase (ALT), creatinine (Scr), blood urea nitrogen (BUN), and glomerular filtration rate (eGFR) were measured by using an automatic biochemical analyzer at the Laboratory Department of Hunan Provincial People's Hospital.

#### Assessment of potential covariates

Education level was divided into four groups: elementary school and below, middle school, high school, and college and above. Marital status was classified as single married. Patients were judged to be exercising regularly based on the number of times they exercised in a week: 0 as no exercise, 1–3 as irregular exercise, and >3 as regular exercise. We defined current smoking as smoking one or more cigarettes per day consecutively or cumulatively for a period of 6 months; Alcohol consumption was currently defined as at least 2 drinks per week.

#### Body composition assessment

The height, weight and waist circumference (WC) of the study subjects were measured by speziell researchers using a height-weight scale while the patients wore light clothing and were barefoot. The average readings after three measurements were used for analysis. BMI = weight (kg)/height (m^2^).

BFP = −44.988+ (0.503 × age) + (10.689 × sex) + (3.172 × BMI) - (0.026 × BMI2) + (0.181 × BMI × sex) - (0.02 × BMI × age) - (0.005 × BMI^2^ × sex) + (0.00021 × BMI^2^ × age), where sex represented 1 for women (8).

We divided the study population into four groups based on the quartiles of BFP: 33.4% or lower (Q1), 33.4–36.1% (Q2), 36.1–39.1% (Q3), and >39.1% (Q4).

#### Definition of H-type hypertension

Patients with H-type hypertension were defined as having a diagnosis of essential hypertension with Hcy≥10 umol/L.

### Statistical methods

Categorical variables were expressed as numbers and percentages (%), and the χ2 test was used to compare differences between groups. Data for continuous variables were expressed as means ± standard deviations (SD), and *t*-test was used to compare differences between groups. A restricted cubic spline was used to examine the relationship between continuous BFP levels and H-type hypertension in postmenopausal women. Multivariate logistic regression models were used to assess the association between BFP quartile subgroups and the risk of H-type hypertension. The logistic regression models included known potential confounders between BFP and H-type hypertension, as well as covariates with *P* < 0.05 in univariate analysis. We constructed three models: model 1 was unadjusted; in model 2, we adjusted for age, BMI, WC, educational background, marital status, exercise, smoking history, and drinking history. Model 3: TG, LDL, Scr, BUN, and eGFR was added to model 2. Stratified analysis was performed for BMI, smoking and drinking history, and exercise.

All statistical analyses were performed using SPSS 26.0 and R 4.2.1, and *P*-values <0.05 were considered statistically significant.

## Results

### Basic information

This study pooled 1,597 postmenopausal women with essential hypertension. The average age of all subjects was 63.1 ± 9.5 years. A total of 955 (59.8%) H-type hypertensive patients were identified in this research. The average BFP of all individuals was 36.3 ± 4.2%, with 397 (24.9%), 401 (25.1%), 399 (24.9%), and 400 (25.1%) in each group following quartile grouping (Table 1). We discovered that the prevalence of H-type hypertension was also highest in the fourth quartile group of BFP (*P* < 0.05). Table 1 also showed a comparison of baseline information between postmenopausal women with and without H-type hypertension events. Obesity, low education, marriage, less exercise, smoking, and drinking were all associated with an increased risk of H-type hypertension compared to the control group. In addition, WC, TG, LDL, Scr, and BUN increased significantly in HHcy patients, while eGFR decreased significantly (*P* < 0.05).

**Table 1:** Baseline characteristics of study participants according to H-type hypertension status.

**Characteristics**	**Total**	**Essential** **hypertension**	**H-type** **hypertension**	**χ^2^/Z**	***P*** **–value**
BMI, *n* (%)				75.6	<0.001
<18.5 kg/m^2^	85 (5.3)	45 (7.0)	40 (4.1)		
18.5–23.9 kg/m^2^	915 (57.3)	439 (68.4)	476 (49.8)		
≥24.0 kg/m^2^	597 (37.4)	158 (24.6)	439 (45.9)		
Education background, *n* (%)				17.9	<0.001
Primary school and below	454 (28.4)	147 (22.9)	307 (32.2)		
Junior school	526 (32.9)	224 (34.9)	302 (31.6)		
Senior school	421 (26.4)	192 (29.9)	229 (23.9)		
College and above	196 (12.3)	79 (12.3)	117 (12.3)		
Marital status, *n* (%)				68.3	<0.001
Single	228 (14.3)	35 (5.4)	193 (20.2)		
Married	1,369 (85.7)	607 (94.5)	762 (79.8)		
Exercise, *n* (%)				48.9	<0.001
No exercise	672 (42.1)	210 (32.7)	462 (48.4)		
Irregular exercise	482 (30.2)	249 (38.8)	233 (24.4)		
Regular exercise	443 (27.7)	183 (28.5)	260 (27.2)		
Smoking history, *n* (%)				4.5	0.037
Never smokes	1,506 (94.0)	615 (95.8)	891 (93.3)		
Current or former smokers	91 (6.0)	27 (4.2)	64 (6.7)		
Drinking history, *n* (%)				5.1	0.023
No	1,542 (96.6)	628 (97.8)	914 (95.7)		
Yes	55 (3.4)	14 (2.2)	41 (4.3)		
BFP, *n* (%)				192.5	<0.001
Q1	397 (24.9)	247 (38.5)	150 (15.7)		
Q2	401 (25.1)	197 (30.7)	204 (21.4)		
Q3	399 (24.9)	130 (20.3)	269 (28.2)		
Q4	400 (25.1)	68 (10.5)	332 (34.7)		
Age (years)	63.1 ± 9.5	58.5 ± 7.2	66.2 ± 9.6	−18.2	<0.001
WC (cm), mean ± SD	85.0 ± 27.3	83.3 ± 9.7	86.2 ± 34.5	−2.4	0.016
TG (mmol/L), mean ± SD	1.6 ± 0.7	1.5 ± 0.7	1.6 ± 0.7	−2.8	0.004
TC (mmol/L), mean ± SD	4.6 ± 1.0	4.5 ± 1.0	4.5 ± 1.0	−0.4	0.719
LDL (mmol/L), mean ± SD	2.7 ± 0.9	2.6 ± 0.	2.7 ± 0.8	−2.2	0.026
HDL (mmol/L), mean ± SD	1.2 ± 0.3	1.2 ± 0.3	1.2 ± 0.3	−0.5	0.625
ALT (U/L), mean ± SD	19.2 ± 10.7	19.2 ± 10.4	19.1 ± 10.8	0.02	0.987
Scr (μmol/L), mean ± SD	60.9 ± 13.7	55.5 ± 11.7	64.4 ± 13.8	−13.8	<0.001
BUN (mmol/L), mean ± SD	5.5 ± 1.7	5.2 ± 1.2	5.71 ± 1.8	−6.2	<0.001
eGFR (ml/min), mean ± SD	89.3 ± 18.5	96.7 ± 13.5	84.4 ± 19.7	−17.7	<0.001

### Dose-response relationship between BFP and H-type hypertension

**Figure 1:**
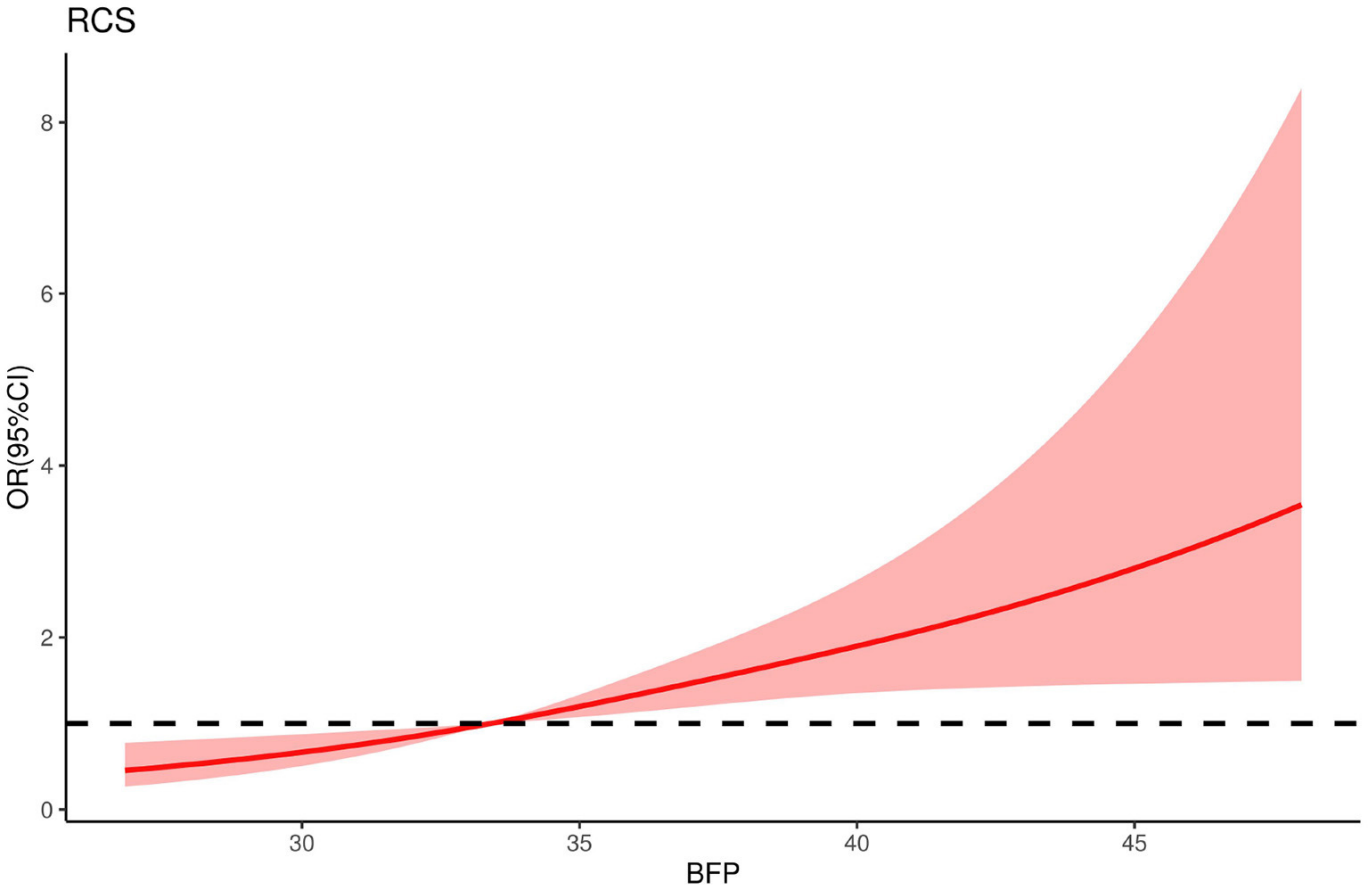
The restricted cubic spline for the relationship between BFP and H-type hypertension in postmenopausal women. The reference value for BFP was set as a cut-off value for the first quartile. Three nodes were selected for the model and adjusted for age, BMI, WC, educational background, marital status, exercise, smoking history and drinking history, TG, LDL, Scr, BUN, and eGFR.

Figure 1 displays a restricted cubic spline model with knots placed at the 5th, 50th, and 90th percentiles to examine the relationship between continuous BFP and the risk of H-type hypertension. The results indicate a significant non-linear dose-response association between BFP and the risk of H-type hypertension after adjusting for confounders. The risk of H-type hypertension in postmenopausal women increased with a continuous increase in BFP.

### Association between BFP and H-type hypertension

Table 2 shows the results of the association between BFP and the risk of H-type hypertension. In the univariate analysis, the OR of BFP and H-type hypertension increased significantly with increasing quartiles of BFP. The OR of quartile 4 was significantly higher than quartile 1 (OR = 7.8, 95% CI: 5.5–11.0; *P* < 0.001). After correction for some confounders in model 3 (age, BMI, WC, educational background, marital status, exercise, history of smoking, history of alcohol consumption, TG, LDL, Scr, BUN, eGFR), the multivariate corrected dominance ratios (95% CI) for the association between BFP and H-type hypertension in the other three groups compared with quartile 1 were 1.2 (0.8–1.7), 1.8 (1.1–3.2), and 3.2 (1.3–7.5), respectively. We also analyzed BFP as a continuous variable. In model 1, each unit increase in BFP was associated with a 0.2-fold increase in the risk of H-type hypertension in postmenopausal women (95% CI: 1.1–1.2; *P* < 0.001). Adjusting for confounders had no effect on this relationship.

**Table 2:** Association between BFP and H-type hypertension in different models.

**BFP (%)**	**Model 1**		**Model 2**		**Model 3**	
	**OR (95%CI)**	***P*** **-value**	**OR (95%CI)**	***P*** **-value**	**OR (95%CI)**	***P*** **-value**
Per 1 unit increase (%)	1.2 (1.1–1.2)	<0.001	1.4 (1.1–1.6)	<0.001	1.3 (1.1–1.6)	0.003
Quartile (%)
Q1 (<33.4)	Reference		Reference		Reference	
Q2 (33.4–36.1)	1.6 (1.2–2.2)	<0.001	1.3 (0.9–1.9)	0.188	1.2 (0.8–1.7)	0.444
Q3 (36.1–39.1)	3.5 (2.6–4.7)	<0.001	1.9 (1.2–3.4)	0.013	1.8 (1. 1–3.2)	0.029
Q4 (>39.1)	7.8 (5.5–11.0)	<0.001	3.2 (1.4–7.8)	0.008	3.2 (1.3–7.5)	<0.001

*Model 1: we did not adjust any confounding factors*.

*Model 2: we adjusted age, BMI, WC, educational background, marital status, exercise, smoking history and drinking history*.

*Model 3: we adjusted for model 2 plus TG, LDL, Scr, BUN, eGFR*.

### Subgroup analysis

The association between BFP and H-type hypertension was analyzed in a predetermined manner according to the following factors: age, BMI, smoking, drinking, and exercise. Table 3 showed that the positive association between BFP and H-type hypertension was maintained across all stratified subgroups (*P* > 0.05 for all interactions).

**Table 3:** Association between BFP and H-type hypertension in different subgroups.

**Subgroups**	**All participants**	**OR (95%CI)**	***P*** **-value**	**P for interaction**
**Age, *n* (%)**				0.970
<60 years old	702	1.1 (1.0–1.2)	0.044	
≥60 years old	895	1.2 (1.1–1.3)	0.003	
**BMI, *n* (%)**				0.480
<18.5 kg/m^2^	85	1.8 (1.2–2.7)	0.005	
18.5–23.9 kg/m^2^	915	1.1 (1.0–1.2)	0.006	
≥24.0 kg/m^2^	597	1.2 (1.1–1.3)	<0.001	
**Smoking history, *n* (%)**				0.807
Never smokes	1,506	1.2 (1.1–1.3)	<0.001	
Current or former smokers	91	1.2 (1.0–1.3)	<0.001	
**Drinking history, *n* (%)**				0.752
No	1,542	1.2 (1.1–1.3)	0.039	
Yes	55	1.3 (1.0–1.4)	<0.001	
**Exercise, *n* (%)**				0.342
No exercise	672	1.5 (1.3–1.8)	<0.001	
Irregular exercise	482	2.1 (1.5–2.7)	<0.001	
Regular exercise	443	1.6 (1.2–2.3)	<0.001	

*Data are adjusted for age, BMI, WC, educational background, marital status, exercise, smoking history, drinking history, education background, TG, LDL, Scr, BUN, eGFR*.

## Discussion

The association between BFP and H-type hypertension in postmenopausal women was investigated in this cross-sectional study. The hazard of H-type hypertension in postmenopausal women was found to be associated with BFP by logistic regression, and higher BFP was more closely associated with H-type hypertension. After reconciling possible confounding factors, we obtained the same results. The positive correlation between BFP and H-type hypertension was constant across all stratified groupings.

H-type hypertension has become a common chronic condition in China in recent years, and it plays a pivotal role in cardiovascular, brain, and renal problems. Patients with H-type hypertension had a 5-fold increase in cardiovascular events compared to hypertension alone (15). A considerable amount of literature has been demonstrated that HHcy and hypertension have a synergistic impact that raises the risk of cardiovascular events (2, 16). Therefore, early prevention is extremely necessary for H-type hypertension. Obesity has been identified as an independent risk factor for H-type hypertension in previous investigations. It has been proposed that the association between hcy and hypertension was influenced by BMI from a meta-analysis (17). However, in another cross-sectional study, a significant association was only observed between BMI and Hcy, but not with hypertension (18). Some researchers speculated that this discrepancy may be since BMI is not accurate as a measure of obesity (19). In Asian populations, BMI may underestimate the role of body fat content BMI (20). Therefore, it may be more reliable to use BFP as an indicator of obesity (21). Takase has demonstrated that it is BFP rather than BMI that affects blood pressure (22). A Korean cohort study similarly showed that increased body fat was a predictor of hypertension (23). Furthermore, BFP played an paramount role in the identification of hypertension in women (24).

Menopause is a life process that every woman must go through, when ovarian function declines and estrogen in the body decreases, resulting in a series of pathophysiological changes in the organism (25). In addition to menopausal-related symptoms, psychiatric and neurological symptoms, postmenopausal women also experience significant changes in the cardiovascular system, especially hypertension (26). Menopause has become an independent risk factor for increased morbidity and mortality of hypertension in women (14). Current studies have also found elevated Hcy in postmenopausal women compared to premenopausal women (27). Relevant epidemiological surveys have indicated that the prevalence of H-type hypertension in postmenopausal women is higher than that in men of the same age and in non-menopausal women, and it tends to increase with age (28). Therefore, H-type hypertension can seriously affect the amount of life dwelling and health level of menopausal women. Also, BFP in women increased at a similar annual rate with age (29). Similar findings were reported in our investigation, demonstrating that BFP is a risk factor for H-type hypertension in postmenopausal women.

The pathogenesis of obesity and H-type hypertension in postmenopausal women is still unclear. A decrease in E_2_ levels may also cause an increase in Hcy levels, leading to endothelial dysfunction in postmenopausal women and increasing the risk of hypertension (30). Furthermore, leptin levels have been observed to be higher in postmenopausal women with hypertension, and obesity has been attributed to alterations in the leptin-activated melano cortical pathway. Meanwhile, bioactive substances secreted by adipose tissue, such as angiotensinogen, IL-6 and TNF-α, are associated with changes in vascular inflammation and arteriosclerosis, leading to increased blood pressure (31, 32).

However, some limitations should be noted. First, the cross-sectional design was not able to accurately assess the causality between BFP and H-type hypertension, and some recall bias possibly existed. Second, we merely evaluated at postmenopausal women, and the findings of our work cannot be extended to other populations. Finally, although we performed multivariate adjustment, we did not exclude other potential confounders.

## Data availability statement

The raw data supporting the conclusions of this article will be made available by the authors, without undue reservation.

## Ethics statement

The studies involving human participants were reviewed and approved by the Medical Ethics Committee of Hunan Normal University (No. 034/2017). The patients/participants provided their written informed consent to participate in this study. Written informed consent was obtained from the individual(s) for the publication of any potentially identifiable images or data included in this article.

## Author contributions

SD: research design, data analysis, and manuscript writing. XH: funding acquisition, conceptualization, and writing—review and editing. YY: data collection and research design. ZD: data collection and comments. TY: data collection and investigation. All authors contributed to the article and approved the submitted version.

## Funding

This study was funded by the National Natural Science Foundation of China (8177120863), Hunan Provincial Science and Technology Department (2020JJ4047), and Changsha Science and Technology Bureau (kq1801096). The funders had no role in the study design, data collection, and analysis, decision to publish, or preparation of the manuscript.

## Conflict of interest

The authors declare that the research was conducted in the absence of any commercial or financial relationships that could be construed as a potential conflict of interest.

## Publisher's note

All claims expressed in this article are solely those of the authors and do not necessarily represent those of their affiliated organizations, or those of the publisher, the editors and the reviewers. Any product that may be evaluated in this article, or claim that may be made by its manufacturer, is not guaranteed or endorsed by the publisher.
